# Spatial Covariance between Aesthetic Value & Other Ecosystem Services

**DOI:** 10.1371/journal.pone.0068437

**Published:** 2013-06-28

**Authors:** Stefano Casalegno, Richard Inger, Caitlin DeSilvey, Kevin J. Gaston

**Affiliations:** Environment and Sustainability Institute, University of Exeter, Penryn, Cornwall, United Kingdom; University of Warwick, United Kingdom

## Abstract

Mapping the spatial distribution of ecosystem goods and services represents a burgeoning field of research, although how different services covary with one another remains poorly understood. This is particularly true for the covariation of supporting, provisioning and regulating services with cultural services (the non-material benefits people gain from nature). This is largely because of challenges associated with the spatially specific quantification of cultural ecosystem services. We propose an innovative approach for evaluating a cultural service, the perceived aesthetic value of ecosystems, by quantifying geo-tagged digital photographs uploaded to social media resources. Our analysis proceeds from the premise that images will be captured by greater numbers of people in areas that are more highly valued for their aesthetic attributes. This approach was applied in Cornwall, UK, to carry out a spatial analysis of the covariation between ecosystem services: soil carbon stocks, agricultural production, and aesthetic value. Our findings suggest that online geo-tagged images provide an effective metric for mapping a key component of cultural ecosystem services. They also highlight the non-stationarity in the spatial relationships between patterns of ecosystem services.

## Introduction

Key to the successful maintenance and management of environmental resources is an understanding of their spatial distribution. Recognition of the significance of, and threats to, ecosystem services (the benefits that humans gain from ecosystems), has thus been associated with growth in attempts to map their patterns of variation and covariation [1]–[4]. In many instances, however, this remains challenging. Of the four main categories of services (supporting, provisioning, regulating and cultural [5]), this is particularly true of cultural services: the non-material benefits people gain from ecosystems (including aesthetic, recreational and spiritual benefits). Therefore, most studies have tended to focus on provisioning and regulating services, rather than cultural ones [6], [7]. Indeed, whilst there has been substantial improvement in understanding of the fundamental importance of cultural services, their effective integration into the application of ecosystem service frameworks has been limited by the challenges of quantifying, valuing and mapping them [8].

Efforts to quantify and map cultural services have concentrated foremost on estimates of the relative numbers of recreational visitors to particular areas [1], [2], [9], [10]. Other measures include the number of tourist attractions, tax value of summer cottages, number of reported sightings of rare species [11], tourist expenditure [12], accessibility to natural areas [13], days spent fishing [14] and indices combining multiple such variables [15]–[17]. When compared, these measures have, in the main, been found to be weakly correlated with spatial variation in other ecosystem services, which has significant implications for identifying and managing priority areas for their maintenance [1], [2], [11]. However, although these data are useful, a much broader portfolio of metrics and proxies for cultural services, and an understanding of how these covary with measures of other ecosystem services, is urgently required.

Quantification of the aesthetic value that people place on different parts of the landscape represents an innovative development in the mapping of cultural services. One potential measure of aesthetic value can be found in the spatial distribution of photographs of the natural environment that people post online, working from the premise that areas more highly valued for their aesthetic attributes will generate ‘hotspots’ of activity. Particularly useful are Internet platforms which specifically facilitate posting of geo-tagged digital images, and which are populated by an increasing number of users worldwide. Here we exploit one such resource, the “Panoramio” web platform (www.panoramio.com), to provide a valuable additional metric of cultural ecosystem services (aesthetic value), and document its relationship with two other ecosystem goods and services: a provisioning service (agricultural production) and a supporting/regulating service (carbon stocks in soil).

## Materials and Methods

The study was carried out in Cornwall, UK, as a regional approach is considered most appropriate for the analysis of cultural activity [18]: tourism and agriculture are the largest components of the economy of this particular region [19]; and both residents and visitors have extensive (albeit not universal) access to digital cameras and the internet [20]–[21] that are required for the use of posted geo-tagged images.

### Aesthetic value (cultural service)

Panoramio hosts photos of “places of the world”, with a particular focus on images of landscapes, natural features (such as woodlands) and animals in their natural environment [22]. Images that have as their central subject people, machines, vehicles or the interiors of structures, or that depict public events such as fairs or concerts, are excluded from the platform [22]. The semantic content of Panoramio's images makes it better adapted to measure the perceived aesthetic value of ecosystems than other geo-tagged web platforms, that do not focus on landscape and environment. The number of individuals per unit area (1 km^2^) uploading photographs to Google Earth via the Panoramio web platform was used as our measure of aesthetic value. This measure is more appropriate than the total number of photographs uploaded in each area, which reflects the level of activity of individual photographers rather than the overall value placed on a site by visitors.

### Soil carbon (supporting and regulating service)

Data on carbon storage in soil (1 km^2^ resolution map) were obtained from the European Commission Joint Research Centre [23], [24]. These data are especially accurate for England, as detailed ground survey verification has been carried out.

### Agricultural production (provisioning service)

Following Anderson et al. [1] and Eigenbrod et al. [25], we calculated an overall measure of agricultural production by summing the gross margins for all major crops/livestock. As inputs we used agricultural census data [26] at ward level, the CORINE land cover map [27], and gross margin estimates [28]. Agricultural production was expressed in units of £ per ha, and processed at 100 m resolution and then resampled at 1 km resolution. We improved on the original methodology by computing an averaged agricultural value from 2000 to 2005 (instead of using one year of data); differentiating gross margins according to lowlands, disadvantaged and severely disadvantaged areas; and using more precise input land cover data to achieve a higher resolution.

### Analysis

Ecosystem service data were normalised to a 0–100 scale for comparison. We tested potential bias of aesthetic value by population density and by coastal/non-coastal locations. Cornish population densities were obtained from the 2011 census [29]. We selected 55 centres including the main towns (population greater than 3,000) and the populations coincident with hotspots of aesthetic value (>40 photographers per grid cell: the upper 99th quantile).

**Figure 1:**
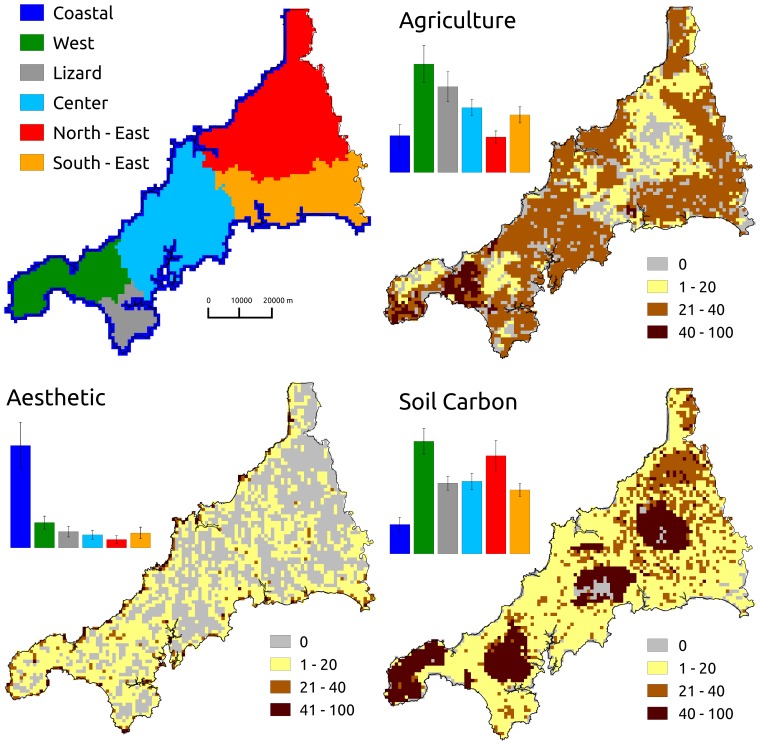
Study area and ecosystem services distribution. Geographical zonation of Cornwall (upper left), the distribution of agriculture, aesthetics and soil carbon (other maps; variation scaled from 0–100), and the mean value of each ecosystem service within each geographical zone (histograms).

Spatially autocorrelated data violate the assumption of sample independence for a traditional test of significance. Therefore, we tested the presence of spatial autocorrelation using Moran's I coefficient [30] and quantified correlations between ecosystem services using the CRH-method [31], [32], which correct correlation statistics for spatial autocorrelation [33]–[35]. Because of the distribution of our data we rank transformed inputs as outlined in Zar (2007) [36] correctly to apply the CRH method [33], [34]. To determine the influence of spatial extent on results, we also divided Cornwall into six separate zones: coastal, Lizard peninsula, west, north-east, south-east and central Cornwall (Figure 1). We performed comparison tests for the overall study area and within separate zones, and corrected results for multiple test significance by applying Benjamin-Hochberg corrections on p-values [37].

The aesthetic and agricultural production surfaces were generated using Bash-Awk and GRASS [38]. Statistical analyses were performed in R [39] using “SpatialPack” and “raster” libraries for spatial related statistics.

## Results

**Figure 2:**
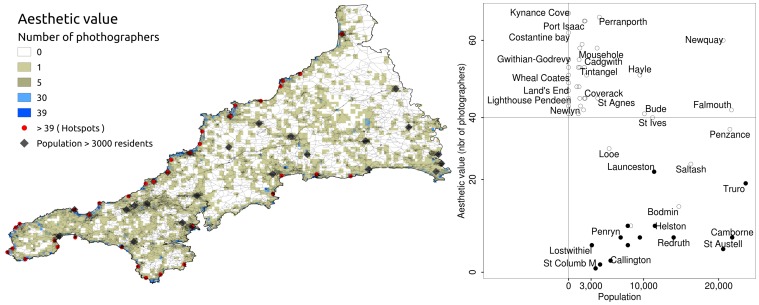
Aesthetic value and population of Cornwall. Aesthetic value map of Cornwall (left) and spatial covariance with the most populated places in Cornwall (right). Population data: Office for National Statistics, mid year estimates 2010. White dots: coastal locations; black dots: inland locations (not all location labels are shown).

A total of 113,686 photographs were uploaded by 15,413 users in Cornwall from 2005 to 2011; 9,632 photographers in coastal, 1,414 in west, 1,411 in north-east, 1,385 in central, 1,227 in south-east and 344 in the Lizard peninsula (Figure 1). Hotspots of aesthetic value (35 of 3,843 grid cells; Figure 2) were all located in coastal areas: seven were in coastal towns (population>3000), 17 close to sparsely populated settlements (<3000 inhabitants), and 11 in unpopulated areas (beaches or touristic coastal sites).

There was a negative correlation between population density and aesthetic value (CRH correlation = −0.56, n = 55, *p-value<0,001*).

Soil carbon storage was highest in four main areas, located in the west and in three inland parts of Cornwall (Figure 1). The zonal statistics showed low carbon storage in coastal areas and highest in west and north-east Cornwall (Figure 1). Agricultural production was highest in west Cornwall and the Lizard peninsula, and lowest in coastal zones and in the north-east region.

**Figure 3:**
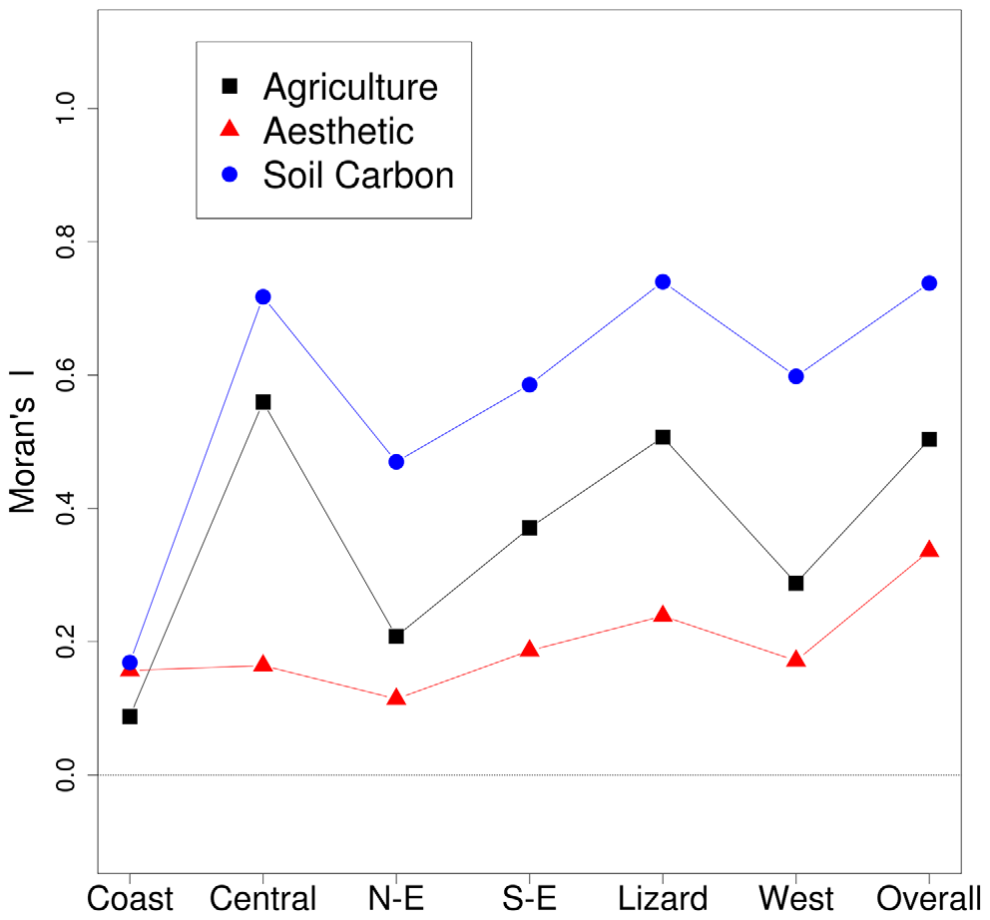
Test of spatial autocorrelation for agriculture, aesthetics and soil carbon data in Cornwall and within each geographical zone.

We found positive spatial autocorrelation in all ecosystem service layers (Figure 3): soil carbon had the highest while aesthetic value had more dispersed spatial patterns. Compared to the overall study area, regional zones such as Coastal, North-East and West Cornwall had weaker patterns of spatial autocorrelation. Across the whole region, agricultural production was negatively correlated with both aesthetic value and soil carbon storage, and the latter two were themselves weakly negatively correlated (Table 1). However, within zones the relationships were quite variable in strength (Table 1).

**Table 1:** CRH correlation test between different ecosystem services across Cornwall, and within different regions thereof.

	Coastal	Central	North-E	South-E	Lizard	West	Overall
**Agriculture vs Aesthetics**							
n	602	419	160	872	1,167	623	3,843
CRH	−0.02	−0.26*	−0.07	−0.07*	−0.12	−0.23*	−0.16*
**Agriculture vs Soil Carbon**							
n	308	417	160	836	1,154	603	3,478
CRH	0.10	−0.52**	−0.03	−0.07*	−0.45***	−0.16***	−0.22***
**Soil Carbon vs Aesthetics**							
n	308	417	160	836	1,154	603	3,478
CRH	0.04	0.06	−0.13	0.03	0.02	0.08	−0.11***

Multiple significance tests adjusted using Benjamin-Hochberg's (Benjamin et al. 1995) corrections: *p-value<0.05; ** p-value<0.01; *** p-value<0.001.

## Discussion

Most mapping of geographic variation in ecosystem services, and analysis of the patterns of covariation between services, has been conducted by ecologists and conservation biologists [6]. This has tended to result in a heavy reliance on conventional data sources with an established geographically explicit component. This is particularly the case for cultural ecosystem services, for which measures have in consequence been quite restricted, and for which novel approaches will need to be explored. Here we present one such approach, using data derived from social media to capture variation in the value that people place on different parts of the landscape.

The emerging field of computational social science exploits the capacity to collect and analyse data about human interactions on an unprecedented scale [40]. The potential for online digital data sets to provide valuable information for scientific studies has been well recognised [41] and applied to diverse disciplines [42]–[47], but to our knowledge they have not previously been employed in the context of ecosystem services. The research shares its approach with diverse computational social sciences applications [42]–[44], [46]–[47] which aim to identify relationships between people's behaviour online and real world quantities.

We find substantial variation in our measure of aesthetic value across the study region. The peaks are distinct from the centres of human population, contrasting with the findings of previous studies that have measured recreational usage [1], [25], but reflecting a priori expectation.

Our findings lend further support to the general conclusion that spatial variation in cultural services tends to be poorly or negatively correlated with that in many other ecosystem services [1], [2], [11], albeit we consider only single supporting/regulating and provisioning services. This would seem to highlight the need both to explore and test a variety of measures of cultural services, and to identify some such measures that are particularly well-founded and robust. Any failure to do so will likely serve to hinder attempts to ensure that cultural services get fuller consideration when planning for the maintenance of ecosystem service provision both at present and in the future.

We also find that the extent over which patterns of covariation in ecosystem services are determined can have a marked influence on the outcome, with these patterns typically becoming much more variable among smaller areas. Such an outcome has previously been documented [1], [3] but over yet larger extents than those examined here. This will make generalizing about the relationships between different services challenging, and raises the spectre that observed relationships between variation in different ecosystems services is highly context-specific. Indeed, one can see an analogous situation arising to that which has developed in the context of attempts to understand spatial relationships between patterns of species richness and environmental variables, in which two schools of thought exist, a prevailing one that continues largely to ignore such complexities and another which focuses on the challenges of statistically handling non-stationary processes [48]–[50].
